# Fusion of Hyperspectral CASI and Airborne LiDAR Data for Ground Object Classification through Residual Network

**DOI:** 10.3390/s20143961

**Published:** 2020-07-16

**Authors:** Zhanyuan Chang, Huiling Yu, Yizhuo Zhang, Keqi Wang

**Affiliations:** 1 College of Mechanical and Electrical Engineering, Northeast Forestry University, Harbin 150040, China; changzhanyuan@shnu.edu.cn (Z.C.); zdhwkq@163.com (K.W.); 2 College of Information, Mechanical and Electrical Engineering, Shanghai Normal University, Shanghai 200234, China; 3 College of Information and Computer Engineering, Northeast Forestry University, Harbin 150040, China; yhl2016@163.com

**Keywords:** CASI hyperspectral imagery, airborne LiDAR, data fusion, residual network, deep learning

## Abstract

Modern satellite and aerial imagery outcomes exhibit increasingly complex types of ground objects with continuous developments and changes in land resources. Single remote-sensing modality is not sufficient for the accurate and satisfactory extraction and classification of ground objects. Hyperspectral imaging has been widely used in the classification of ground objects because of its high resolution, multiple bands, and abundant spatial and spectral information. Moreover, the airborne light detection and ranging (LiDAR) point-cloud data contains unique high-precision three-dimensional (3D) spatial information, which can enrich ground object classifiers with height features that hyperspectral images do not have. Therefore, the fusion of hyperspectral image data with airborne LiDAR point-cloud data is an effective approach for ground object classification. In this paper, the effectiveness of such a fusion scheme is investigated and confirmed on an observation area in the middle parts of the Heihe River in China. By combining the characteristics of hyperspectral compact airborne spectrographic imager (CASI) data and airborne LiDAR data, we extracted a variety of features for data fusion and ground object classification. Firstly, we used the minimum noise fraction transform to reduce the dimensionality of hyperspectral CASI images. Then, spatio-spectral and textural features of these images were extracted based on the normalized vegetation index and the gray-level co-occurrence matrices. Further, canopy height features were extracted from airborne LiDAR data. Finally, a hierarchical fusion scheme was applied to the hyperspectral CASI and airborne LiDAR features, and the fused features were used to train a residual network for high-accuracy ground object classification. The experimental results showed that the overall classification accuracy was based on the proposed hierarchical-fusion multiscale dilated residual network (M-DRN), which reached an accuracy of 97.89%. This result was found to be 10.13% and 5.68% higher than those of the convolutional neural network (CNN) and the dilated residual network (DRN), respectively. Spatio-spectral and textural features of hyperspectral CASI images can complement the canopy height features of airborne LiDAR data. These complementary features can provide richer and more accurate information than individual features for ground object classification and can thus outperform features based on a single remote-sensing modality.

## 1. Introduction

With the continuous development of remote sensing technologies, higher quality satellite and aerial images can be obtained. Such images show ground objects with better clarity and improved structural details, thus representing an important data source for remote sensing applications. In particular, hyperspectral image data has dozens or even hundreds of spectral bands, which can provide a wealth of spectral information for remote-sensing applications, especially feature classification [1]. Moreover, airborne light detection and ranging (LiDAR) measurements can quickly lead to three-dimensional surface data, generate three-dimensional coordinates, create a digital surface model, and construct a digital elevation model (DEM) in addition to other characteristic models [2]. Airborne LiDAR systems show strong anti-interference, penetrability, and timeliness capabilities. Moreover, these systems provide a new source of data for the analysis of ground features [3,4].

On the one hand, while hyperspectral imagery shows detailed spectral information, this type of imagery has limitations when discriminating objects with similar spectral characteristics [5,6]. On the other hand, although an airborne LiDAR system can obtain high-precision three-dimensional vertical structure information, such a system cannot accurately classify ground objects due to the lack of corresponding spectral information [7,8]. Hyperspectral data and airborne LiDAR data have been fused in earlier methods to achieve complementary advantages and make up for the individual deficiencies of each technique. This fusion approach led to significant contributions for feature extraction [9]. Chu et al. integrated hyperspectral and LiDAR features using the minimum noise fraction (MNF) transform and the principal component analysis (PCA). Furthermore, the integrated features were used to train a support vector machine classifier for extracting land information in mountain areas [10]. The obtained experimental results showed that multi-sensor data fusion method outperformed methods based on hyperspectral images alone, with an overall accuracy (OA) ranging from 83% to 91%.

In 2018, Dalponte [11] proposed a prediction model of breast height diameters and single-tree crown biomasses using hyperspectral and airborne LiDAR data. In this model, airborne LiDAR data was used to estimate the height and diameter of a single crown, while hyperspectral data was used to identify trees. The results showed that the proposed model had a high accuracy in each of the two prediction tasks. In 2020, Jahan et al. [12] proposed a dual-stream feature fusion method that integrated features from hyperspectral images and LiDAR data for land cover classification, where inverse coefficients were used for feature extraction. The experimental results showed that this method performed well with limited training samples. In this work, we combined features extracted from airborne LiDAR data and hyperspectral compact airborne spectrographic imager (CASI) data [13]. For the hyperspectral CASI images, features of the normalized difference vegetation index (NDVI) and the gray-level co-occurrence matrix (GLCM) were calculated [14,15]. These features were combined with the canopy height model (CHM) of airborne LiDAR data to obtain the surface features of the observed area [16,17].

In recent years, deep learning has emerged as a powerful methodology for feature extraction and classification [18]. The advantages of deep learning in its various forms has also led to remarkable classification performances by operating directly on hyperspectral input [19,20], which effectively solves the problems of traditional supervised classification algorithms (such as Bayesian, Maximum Likelihood, Parallelepiped Classification Method, etc.) depending on suitable samples and the enormous computing problems of high dimensional hyperspectral imagery [21]. Notably, convolutional neural networks (CNN) have achieved good classification results in hyperspectral terrain classification. Hu et al. [22] employed a CNN model to classify hyperspectral features. In this model, CNN local connections, weight sharing, and other enhancements were exploited. These enhancements resulted in significantly reduced model parameters, lower training cost, and improved classification performance. Zhao et al. proposed a multi-scale CNN architecture to extract spatially-relevant depth features for hyperspectral image classification. This architecture led to significantly higher classification accuracy in comparison to traditional methods, especially for urban areas [23]. However, the classification based on this multi-scale architecture requires the selection of different feature extraction scales for different types of ground objects. Moreover, the two-dimensional CNN architecture can only extract separate spatial and spectral image information and does not make full use of the joint spatio-spectral information of hyperspectral images. Zhong et al. [24] designed an end-to-end residual network for hyperspectral image classification. In this network, the original 3D data blocks were used as inputs while the residual blocks were used to learn discriminative hyperspectral features. The results showed that this residual network achieved high classification accuracies on images of agricultural, urban, and rural areas [25]. Though the combination of hyperspectral and airborne LiDAR data has rich and complex spectral, textural, and elevation information, the employment of this information for feature selection and extraction is quite challenging [26], as not all the measurements are significant and useful, and the original feature space may not be the most effective space for representing the data [27]. Therefore, we propose in this paper a hierarchical-fusion multiscale dilated residual network to classify ground objects based on fused hyperspectral CASI and airborne LiDAR features. Particularly, the proposed network effectively extracted rich multi-sensor fused features and fully exploited them for high-accuracy ground object classification.

In this paper, the CASI aerial hyperspectral image and airborne LiDAR data of Zhangye agricultural area in the middle reaches of the Heihe River in China were used as the research objects. We aimed at fusing the CASI hyperspectral image and airborne LiDAR data, using residual neural network to classify the ground object data and effectively improve the ground object classification accuracy, as well as reduce the computational complexity.

## 2. Materials and Methods

### 2.1. Multi-Sensor Data Collection

**Figure 1:**
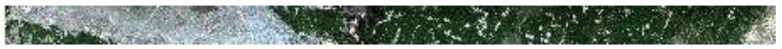
Compact airborne spectrographic imager (CASI) (122642) flight strip image of the Heihe River Basin.

The CASI hyperspectral remote-sensing image data was collected in the visible and near-infrared ranges for the Heihe River eco-hydrological remote-sensing experiment (Hiwater). Geometric, radiometric, and atmospheric correction operations were applied to the raw data. Then, the reflectance of each land cover type was obtained using atmospheric data measurements with synchronous reference to the ground. The CASI image had a spatial resolution of 1.0 m, 48 spectral bands, and a spectral range between 0.38 and 1.05 µm. Figure 1 shows a CASI (122642) flight strip image of the core observation area in the middle reaches of the Heihe River.

**Figure 2:**
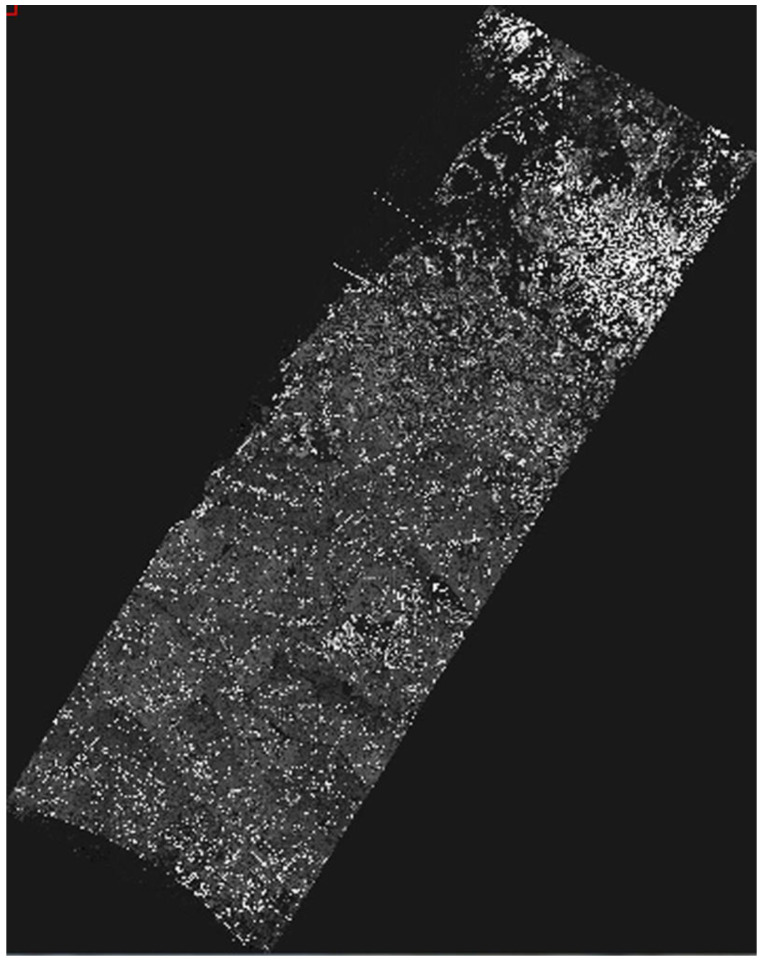
Canopy height model (CHM) data of the Heihe River Basin based on the airborne light detection and ranging (LiDAR) system.

For remote sensing of the eco-hydrological environment in the Heihe River area, an airborne LiDAR experiment was carried out in this area. The flight altitude of the employed aircraft was 2700 m and the laser wavelength of the employed ALS70 LiDAR sensor was 1064 nm. Multiple echoes were recorded and the average point cloud density was 4 points per square meter. The airborne LiDAR aerial data was subjected to several operations: parameter verification and correction, automatic point-cloud filtering and classification, manual editing, as well as other operations. These operations resulted in the generation of a digital surface model (DSM), a digital elevation model (DEM), and a point-cloud density map. Subsequently, the DSM was directly subtracted from the DEM to obtain the canopy height model (CHM) of the middle reaches of the Heihe River Basin. These models provide surface elevation information of high spatial resolution (of the meter level) and high accuracy (20 cm). The airborne LiDAR aerial CHM data is shown in Figure 2.

**Figure 3:**
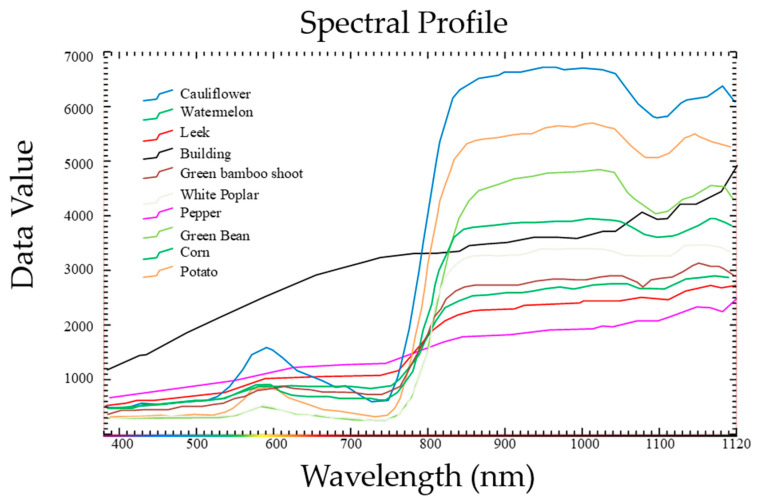
Average spectral curves for different land cover types in the data collection period.

**Figure 4:**
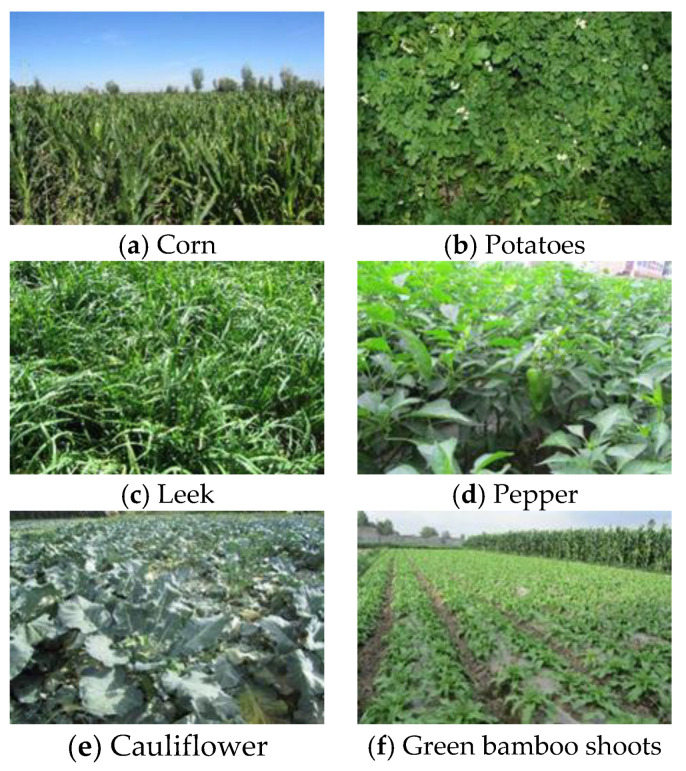
Ground object types in the data collection period.

The observed area had more than 10 kinds of land cover types, such as ‘corn’, ‘leek’, ‘cauliflower’, ‘pepper’, ‘potatoes’, and so on. Ground object classification was highly challenging because of this high diversity of the crop types in the field and the imbalance of the land cover types. Figure 3 and Figure 4 demonstrate the average spectral curves of the land cover types and the ground object types for the same data collection period, respectively.

### 2.2. Image Registration and Dimensionality Reduction

The hyperspectral CASI data and the airborne LiDAR data were collected under different imaging principles by different airborne sensors whose aircrafts fly at different orbits. These differences resulted in disparities in the spatial attributes and coordinate-system inconsistencies among the two resulting images. Image registration was applied before data fusion to account for these inconsistencies and to align the images together.

**Figure 5:**
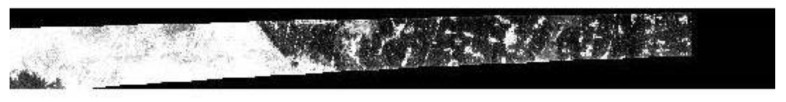
Hyperspectral CASI image after registration.

Using the Image Registration Workflow toolbox in the ENVI software, 10 seed points were manually selected for registration. The CHM point-cloud data map was selected as the reference Base Image File, while the hyperspectral CASI image was selected as the Warp Image File to be registered. The registration parameters selected through the Tie Point Generation panel are listed in Table 1. The registration result is shown in Figure 5.

The minimum noise fraction (MNF) transform can reduce the effects of hardware devices (such as sensors) and processing operations (such as image analysis of raw image data) on hyperspectral images, so that most of the hyperspectral information is concentrated in a few bands and the computational cost is reduced [28,29]. The MNF transform is essentially composed of two PCA transforms in series. The first PCA transform separates and readjusts the input image noise, minimizes the variance of the readjusted noise, and reduces the inter-band correlation. In the second PCA transform, the bands are arranged in a descending order in terms of the signal-to-noise ratio (SNR). Bands with lower SNR have increased noise and less image information.

The computational steps of the MNF transform are as follows. Firstly, the raw hyperspectral image is filtered to obtain a noise covariance matrix *C_N_*, which is then diagonalized as

(1) $$D_{N}=U^{T}C_{N}U$$

where *D_N_* is a diagonal matrix arranged in a descending order of the eigenvalues of *C_N_* and *U* is an orthogonal matrix composed of the corresponding eigenvectors. Based on Equation (1), *P^T^C_N_P* = *I*, where *P = UD_N_^ − 1/2^*, and *I* is an identity matrix. Let the hyperspectral data be *X*, where *X* can be transformed into a new space by a *Y* = *PX* transformation. The noise in *Y* has unit variance and no correlation between bands.

Secondly, we used the constructed matrix *p* to carry out a standard PCA transformation on the total covariance matrix CD of X, so that the matrix after noise processing is $C_{D-adj}=P^{T}C_{D}P$. Subsequently, we have:

(2) $$D_{D-adj}=V^{T}C_{D-adj}V$$

**Figure 6:**
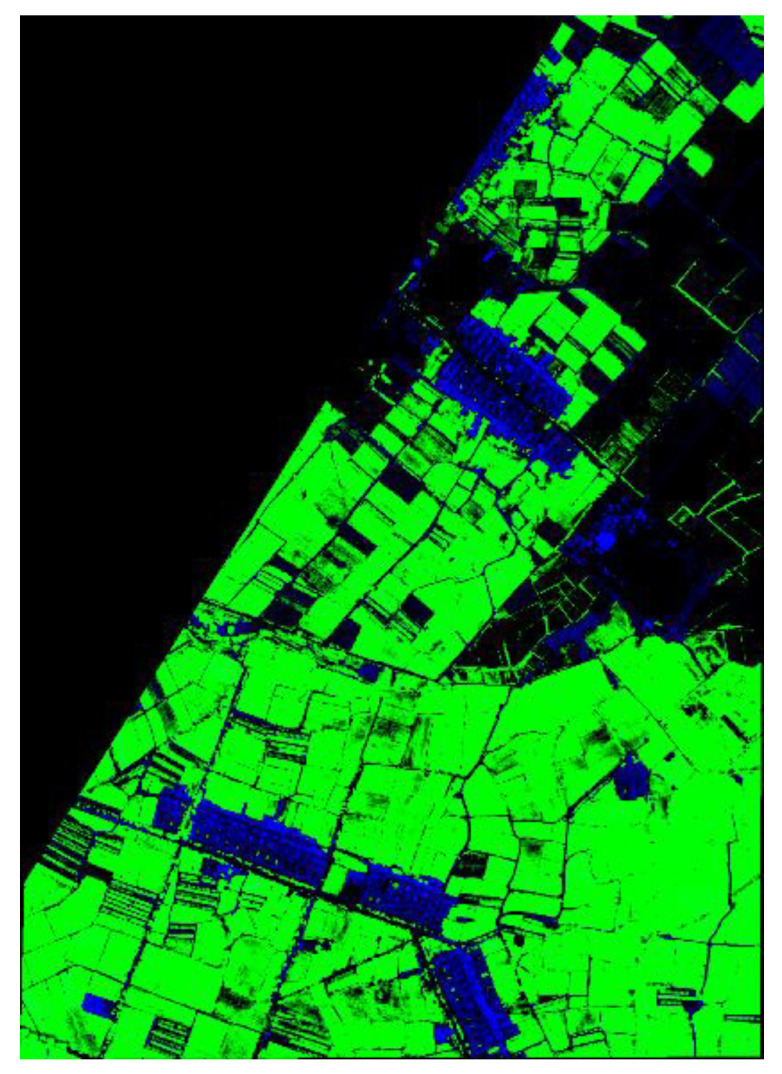
The composite image after the minimum noise fraction (MNF) transform.

In Equation (2), *V* is an orthogonal matrix of eigenvectors and the MNF transform can thus be obtained as $T_{MNF}=PV$. The composite image after the MNF transform is shown in Figure 6.

### 2.3. Feature Extraction

#### 2.3.1. Normalization of the Vegetation Index

**Figure 7:**
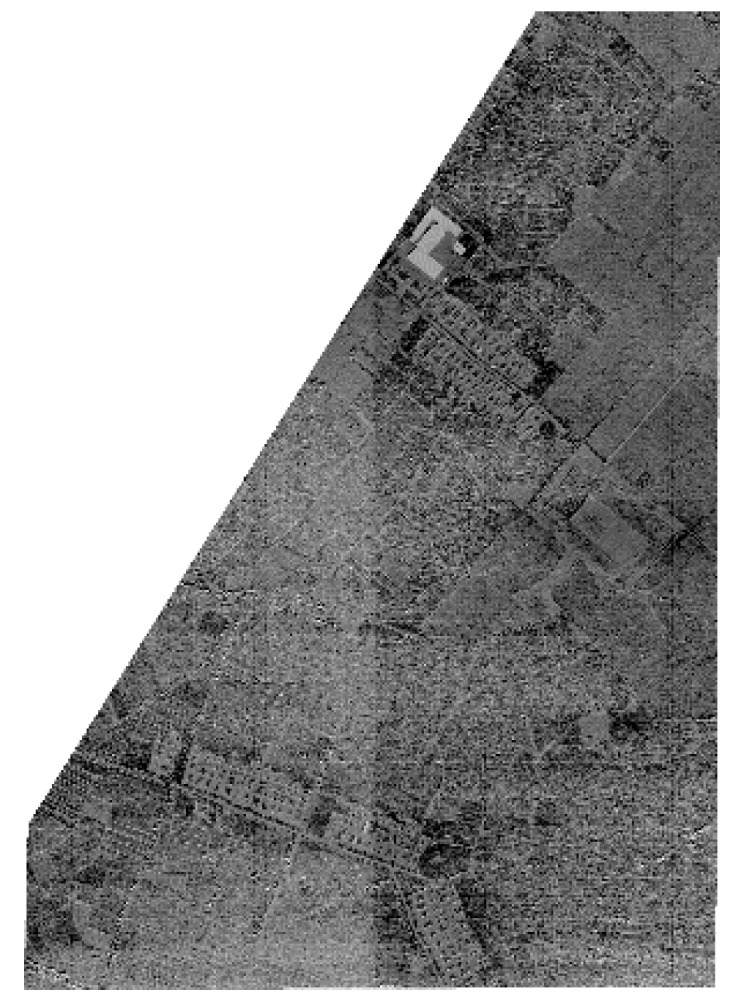
The normalized difference vegetation index (NDVI) map of the observed area.

Ground objects show distinctive morphological characteristics in the infrared and near infrared bands. Specifically, the infrared band shows strong light absorption, while the near infrared band exhibits strong light reflectivity and projection. Hence, variants of the vegetation index can be calculated from these two bands. In fact, the normalized difference vegetation index (NDVI) is positively correlated with vegetation coverage and is thus widely used in the classification of remote-sensing imagery to reflect vegetation growth while effectively reducing the impact of topographic factors. Moreover, the NDVI describes spectral vegetation characteristics, shows good stability, and is simple to calculate. This index is mathematically given by:

(3) $$NDVI=(DN_{NIR}-DN_{R})/(DN_{NIR}+DN_{R})$$

where $DN_{NIR}$ and $DN_{R}$ are the gray-level image intensity values of the near-infrared and red-light bands, respectively. The NDVI range is [−1, 1]. On the one hand, negative NDVI values possibly indicate ground occlusion by clouds or water resources. On the other hand, positive NDVI values indicate that the ground is covered by vegetation, where larger NDVI values correspond to denser vegetation. Otherwise, a zero NDVI value means that the ground may be covered by rocks. Although the NDVI value can generally represent the ground vegetation coverage, the sensitivity of this index for high-density vegetation is low. In addition, according to the wavelength range, the first wavelength band is selected as the red band and the 32nd wavelength band is selected as the near-infrared band. The NDVI map of the observed area is shown in Figure 7.

#### 2.3.2. Gray-Level Co-Occurrence Matrices

Texture reflects the grayscale distribution of pixels and their surrounding spatial neighbors in an image. Indeed, surface characteristics of remote-sensing imagery can be well described using texture features. One of the key families of texture descriptors is based on gray-level co-occurrence matrices (GLCM). Such a matrix is defined over an image as the distribution of co-occurring grayscale or color pixel values at a given offset and a given direction. The GLCM distribution characteristics are typically summarized by second-order features, which can reflect the image clarity, regional contrast, grayscale uniformity, granularity, and other texture information.

**Figure 8:**
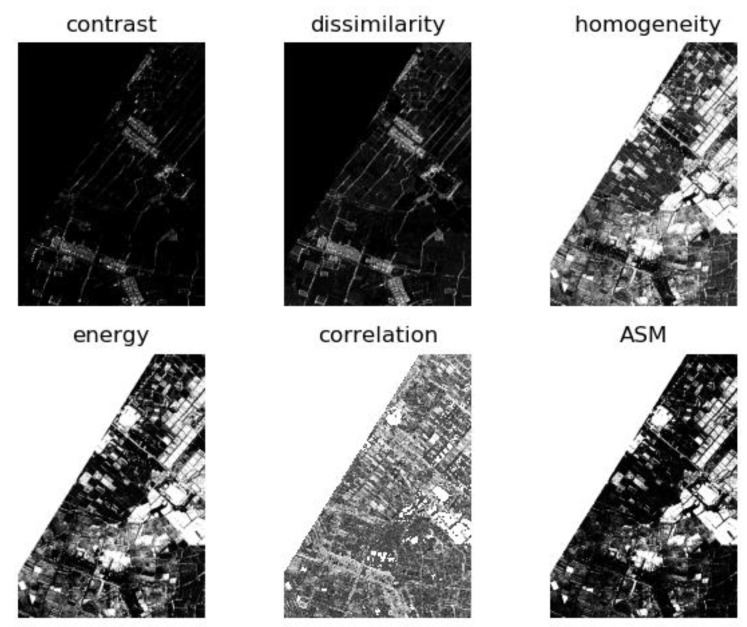
Second-order gray-level co-occurrence matrix (GLCM) features of hyperspectral image data.

In this paper, six second-order GLCM features were computed, namely the contrast, dissimilarity, homogeneity, entropy, correlation, and the angular second moment (ASM). The GLCM window size was set to be 3 × 3 and the texture features of the hyperspectral image data were obtained, as shown in Figure 8.

### 2.4. Ground Object Classification with Hierarchical-Fusion Multiscale Dilated Residual Networks

In this paper, the key spectral bands of the hyperspectral CASI image were identified and the NDVI and GLCM image features were calculated. The hyperspectral features were fused with the CHM airborne LiDAR data. The fused features contained spectral, spatial, textural, and canopy height features from both data sources. Specifically, each fused feature vector had three MNF features, one NDVI feature, six GLCM features, and one CHM feature.

For image classification, a residual network may have deep network layers in order to boost classification performance. In general, different network layers can learn different features: a shallow network layer extracts low-level image details, while a deep network layer can extract high-level image information. In this work, we used a residual network to extract multi-sensor features of remote sensing images, fused the extracted shallow and deep features, made full use of these features, and obtained enhanced ground object classification performance for complex images [30].

**Figure 9:**
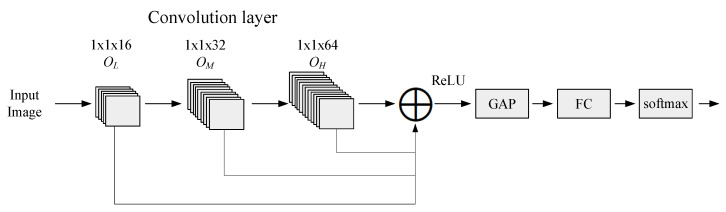
A block diagram of the multi-layer hierarchical fusion residual network for ground object classification.

Figure 9 shows the architecture of the proposed hierarchical fusion residual network. This network was divided into three modules whose outputs were OL, OM, and OH, respectively. These modules have 16, 32, and 64 convolution kernels, respectively. Feature maps of different dimensions were output to realize layer-by-layer feature extraction. In order to ensure dimensionality matching before feature fusion, 64 convolutions were performed with 1×1 convolution kernels. Then, the outputs of the three modules were added and fused. The fused features are mapped by rectified linear unit (ReLU) activation functions and then global average pooling (GAP) were applied [31]. The final feature vectors were generated by inputting the features to fully-connected layers. Finally, a fused feature graph was converted into an output feature vector through the fully-connected network. The output feature vectors were fed to a classifier for ground object classification.

## 3. Experiments and Analysis

### 3.1. Feature Fusion Experiments

In feature fusion experiments, 25 × 25 samples were extracted from the input images. The samples were then divided into training and test sets with 70% and 30% of the samples, respectively. The learning phase was carried out with a learning rate of 0.01, a learning momentum of 0.9, a weight delay of 0.0001, and a maximum number of iterations of 100. The numbers of the training and test samples for various ground objects are listed in Table 2.

In order to assess the effectiveness of the fusion of the hyperspectral CASI data and the airborne LiDAR data, five fusion methods of the respective features were designed, as shown in Table 3.

**Figure 10:**
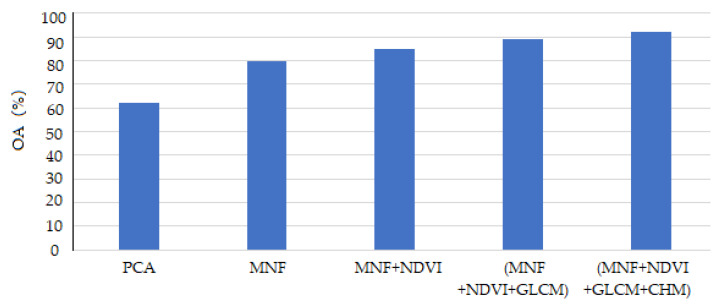
The overall ground object classification accuracy for different feature fusion methods.

The overall accuracy (OA) values obtained by different feature fusion methods are shown in Figure 10. The results show that the OA value for classification with the MNF features was clearly better than that with single-band PCA. This shows the effectiveness of the spectral features extracted from the key spectral bands of the hyperspectral CASI image based on the MNF transform. The third feature fusion method combined the MNF features with the spatio-spectral features of the hyperspectral CASI image. The fourth feature combination augmented the third combination with textural features of that hyperspectral image. The third and fourth combinations led to significantly improved OA values, indicating that the NDVI and GLCM features can be highly distinctive in ground object classification. The fourth feature combination was augmented by the CHM information to form the fifth feature combination, which demonstrates that the CHM data can improve the discrimination of ground objects with different heights. Therefore, the fused features can fully exploit the hyperspectral and LiDAR data and obtain complementary information from these data sources for ground object classification.

The experimental results have shown that classifiers based on a single source of remote sensing data have clear limitations including relatively low accuracy, ambiguity in target interpretation, and limited applicability. Data fusion can effectively exploit the complementary nature of the multi-band information of hyperspectral CASI images and the canopy height information of airborne LiDAR data, reduce the uncertainty of single-source classification methods, increase the applicability of remote sensing systems, and improve the ground object classification accuracy.

### 3.2. Classification with Hierarchical-Fusion Residual Networks

**Figure 11:**
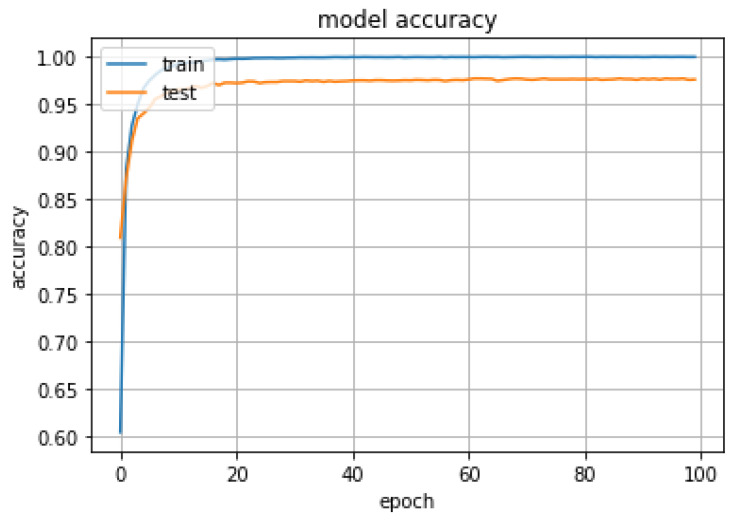
Ground object classification accuracy versus the number of iterations for a hierarchical fusion residual network with a sample size of 25 × 25 and a network depth of 32.

**Figure 12:**
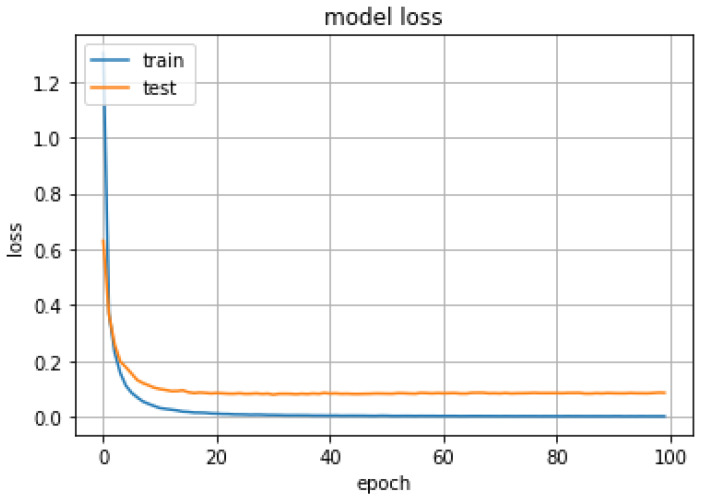
Ground object classification loss versus the number of iterations for a hierarchical fusion residual network with a sample size of 25 × 25 and a network depth of 32.

The fused MNF, NDVI, GLCM, and CHM features extracted from the hyperspectral CASI image data and airborne LiDAR point-cloud data were used as input samples for classifier training and testing. The proposed hierarchical fusion residual network was used for ground object classification. The experiments were setup in the Caffe framework on a computer with a Linux operating system. Figure 11 and Figure 12 show accuracy and loss curves as functions of the number of iterations with a sample block size of 25 × 25 and a network depth of 32. The accuracy clearly increased while the loss dropped substantially. Both measures stabilized after 20 iterations.

As shown in Figure 11 and Figure 12, when the 20th training epoch was reached, the accuracy reaches its maximum value, and then stabilized at about 98%. The loss function value also decreased to its minimum value at the same time. The experimental results show that due to residual block training, the input was directly transferred to the output, information integrity was protected, and performance degradation was highly eliminated.

The ground object classification accuracy based on multi-scale dilated residual networks (M-DRN) is shown in Table 4. Here, OA denotes the overall accuracy, AA denotes the average accuracy, and Kappa denotes the index of agreement or association, which is called the Kappa coefficient. Clearly, the user accuracy (UA) values of all objects can reach more than 97%. Moreover, the classification performance was good for the green bamboo shoots and green beans with few samples, and for the buildings with complex spectral characteristics. The overall accuracy (OA) and the Kappa coefficient reached 97.89% and 0.976. These results demonstrate the effectiveness of fusing the output features of different network levels with high stability.

The block size and the number of layers represent important network parameters. In fact, the sample block size clearly affects the classification accuracy: if the sample block is too large, some details will be ignored; if the sample block is too small, redundant information will be amplified and the classification accuracy will be reduced. Increasing the network depth can improve the classification accuracy to some extent. However, too many network layers may lead to over-fitting, gradient vanishing, or gradient explosion. In this work, these two parameters were set through alternating iterative experiments. In these experiments, the block size was varied between 17×17 and 31 × 31, while the network depth was varied between 12 and 40.

**Figure 13:**
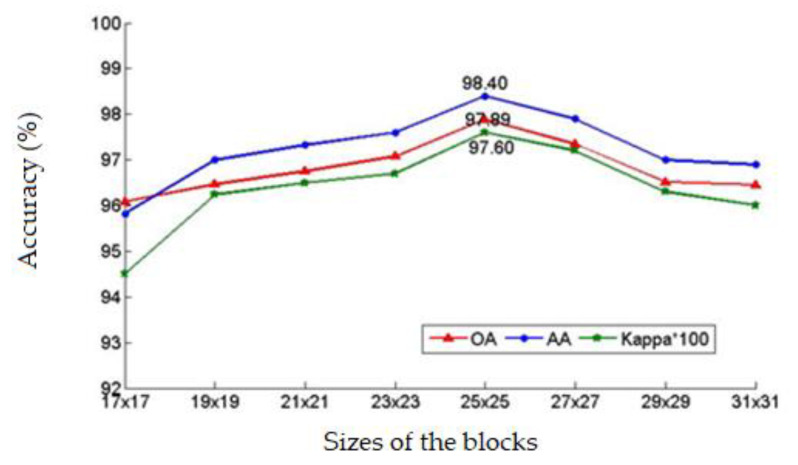
The influence of different block sizes on classification accuracy.

Figure 13 exhibits the variation of the overall accuracy, the average accuracy, and the Kappa coefficient with the increase in the sample block size with a network depth of 32. Obviously, the classification accuracy initially increased, reached a maximum at 25 × 25, and then decreased with the increase of the sample block size. Figure 13 also shows that the sample block size has a certain impact on the classification accuracy in hierarchical fusion residual networks. The result shows that appropriate block size selection as 25 × 25 was important for optimizing classification performance.

**Figure 14:**
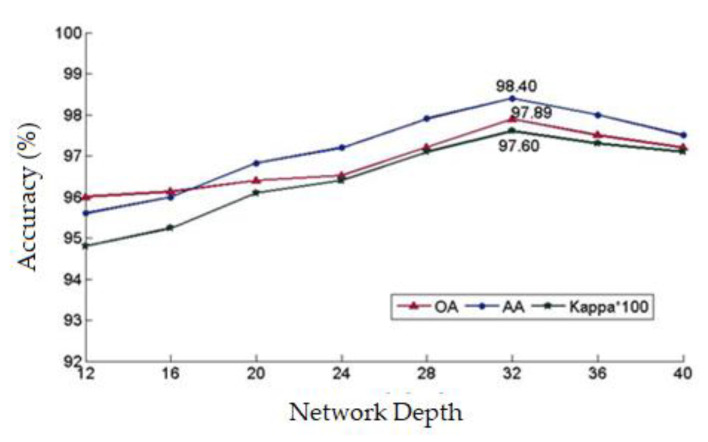
The influence of different network depths on classification accuracy.

Figure 14 shows the variation of the overall accuracy, the average accuracy, and the Kappa coefficient with the increase in the network depth for a sample block size of 25 × 25. Clearly, with the increase of network depth, the classification accuracy steadily increased, reached a maximum at a network depth of 32, and then started to decline, and the trend of changing was consistent with the three parameters. The M-DRN classification algorithm alleviated the problem of performance degradation with increasing network depth. However, when the network depth was excessively high, over-fitting occurred and the classification accuracy declined to a certain degree. Therefore, M-DRN classifiers can improve the overall classification accuracy by deepening the network to a certain limit.

### 3.3. Comparative Classification Experiments

In order to verify the superiority of the M-DRN classifier, this classifier was compared with the SVM (Support Vector Machine), CNN, and DRN classifiers. The SVM classifier took as an input the fused features of the hyperspectral CASI and airborne LiDAR data, using a radial-basis function (RBF) kernel. The classification results of all classifiers are shown in Table 5.

Table 5 shows that the OA value with CNN-based features of the fused image exceeds that of the hand-crafted features and the SVM classifier by 7.44%. The DRN classifier alleviated the network depth burden, extracted deeper features of the fused image, and clearly outperformed the CNN classifier. Comparing the DRN and M-DRN classifiers, we see that the hierarchical fusion mechanism fused complementary and related information of different convolution layer outputs, and learned distinctive features for classification. For the M-DRN classifier, the OA, AA, and Kappa coefficient values exceeded those of the DRN classifier by 5.68%, 5.87%, and 0.058, respectively. The small difference between the OA value and AA value of each classifier also proves that the designed fusion feature had good stability. These results show that the proposed M-DRN classifier has great potential for ground object feature extraction and classification.

**Figure 15:**
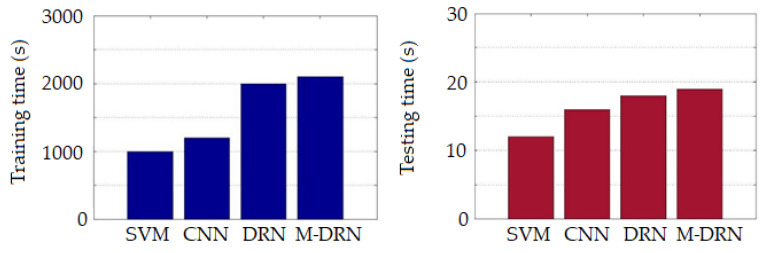
Operation time comparison for different network architectures.

The training and testing times for different network architectures are shown in Figure 15. The training times of the SVM and CNN classifiers with shallow layers were relatively small. Since the DRN classifier had a deeper network architecture, the training time increased. The M-DRN classifier outperformed the DRN one at the cost of a small increase in the computation time. Thus, the M-DRN algorithm remained feasible for practical purposes. While different network architectures can have significantly different training times, they have little differences in terms of the testing time. The network depth (and other several factors) can have noticeable effects on the training time.

## 4. Conclusions

In this paper, hyperspectral images were used to obtain distinctive spectral, spatial, and textural features. Moreover, this work emphasizes the fusion of multi-sensor remote sensing features including hyperspectral image features and the canopy height model features of airborne LiDAR data. The fusion was carried out based on a pixel-level fusion algorithm. These fused features were used to train a residual network for ground object classification.

The key spectral bands of the hyperspectral CASI image data were obtained by the minimum noise fraction (MNF) transform. In addition, gray-level co-occurrence matrices and the normalized difference vegetation index were used to extract textural and spatio-spectral features of hyperspectral images. Then, these features were combined with the canopy height features of airborne LiDAR data to form a multi-source feature set for pixel-level fusion. The SVM, CNN, DRN, and M-DRN algorithms were used to classify the fused features. The experimental results show that the joint advantages of the hyperspectral CASI and airborne LiDAR data were fully utilized by the proposed architecture, and that the addition of different feature types can improve the classification accuracy for different ground objects. Therefore, with limited time and computational resources, the most distinctive features should be selected for different types of ground objects, in order to obtain the best classification performance. Classification methods based on the hyperspectral CASI and airborne LiDAR data are becoming more mature and shall be widely and extensively studied in future land-use information extraction schemes.

In addition, fused hyperspectral and airborne LiDAR features include spatial, spectral, textural, canopy height, and other features. We proposed the use of these features to improve the ground object classification accuracy. However, such an improvement comes at the cost of a large amount of computations. Moreover, while neural network architectures are mainly used here for image classification, whether such architectures are suitable for large size, pixel-level remote sensing image classification needs additional investigations.

## Figures and Tables

**Table 1:** Parameter settings for the ENVI Image Registration Workflow.

Parameter	Setting
Matching algorithm	Mutual information
Minimum tie-point matching threshold	0.01
Geometric model	Fitting global transform
Reference-image registration band	1
Target-image registration band	20
Search window size	128 × 128
Match window size	61 × 61

**Table 2:** The numbers of the training and test samples for various ground objects.

No.	Category	Training/Testing Pixels	Total Pixels
1	Corn	426495/182784	609279
2	Leek	17468/7487	24955
3	White poplar	75211/32233	107444
4	Cauliflower	50467/21628	72095
5	Pepper	107099/45900	152999
6	Potato	18309/7847	26156
7	Green bamboo shoot	3618/1550	5168
8	Watermelon	28318/12136	40454
9	Green bean	1830/785	2615
10	Building	172774/31189	203963

**Table 3:** Different feature fusion methods and numbers for ground object classification.

No.	Fused Features	Number
1	Single-Band PCA	1
2	MNF	3
3	MNF + NDVI	4
4	MNF + NDVI + GLCM	10
5	MNF + NDVI + GLCM + CHM	11

**Table 4:** Ground object classification accuracy based on multi-scale dilated residual networks (M-DRN).

Category	Corn	Leek	White Poplar	Cauliflower	Pepper	Potato	Green Bamboo Shoot	Watermelon	Green Bean	Building
UA (%)	98.92	98.75	99.10	98.54	98.46	98.18	97.63	98.30	97.38	98.77
OA (%)	97.89			AA (%)	98.40			Kappa	0.976	

**Table 5:** Performance metrics for ground object classification using the fused features and each of the SVM, CNN, DRN, and M-DRN classifiers.

	SVM	CNN	DRN	M-DRN
OA (%)	80.32	87.76	92.21	97.89
AA (%)	80.76	87.97	92.53	98.40
Kappa	0.797	0.872	0.918	0.976
